# Supplementary material for: Incidence, Clinical Features and Vaccination Coverage of Pneumococcal Disease in People Living with HIV: A Retrospective Cohort Study (2015–2024)
Source: Vaccines (Basel). 2025 Dec 13;13(12):1240. doi: 10.3390/vaccines13121240 (PMC12737652; doi:10.3390/vaccines13121240)
Supplement: Supplementary file 1 [file vaccines-13-01240-s001.zip › vaccines-3990245-supplementary.pdf]

Supplementary Table S1: *Baseline sociodemographic characteristics and baseline viro-immunological status comparison by sex at birth*

		**Male**<br>**N=7564**	**Female**<br>**N=1083**	**p-value**
**Mode of HIV transmission**	IDU<br>Other	565 (8%)<br>6881 (92%)	211 (20%)<br>856 (80%)	**< 0,001**
**Place of birth**	Spain<br>Other	3197 (45%)<br>3922 (55%)	757 (72%)<br>300 (28%)	**< 0,001**
**CD4 nadir (cells/mm^3)**		338<br>(200 - 510)	210<br>(102 - 329)	**< 0,001**
**HIV peak VL (copies/mL)**		36010<br>(199 - 187100)	51189<br>(5959 - 240652)	**< 0,001**
